# Optimal search strategies for detecting cost and economic studies in EMBASE

**DOI:** 10.1186/1472-6963-6-67

**Published:** 2006-06-06

**Authors:** R James McKinlay, Nancy L Wilczynski, R Brian Haynes

**Affiliations:** 1 Health Information Research Unit, Department of Clinical Epidemiology and Biostatistics, McMaster University Medical Centre, 1200 Main Street West, Room 3H7, Hamilton, Ontario, Canada; 2 Department of Medicine, Faculty of Health Sciences, McMaster University, 1200 Main Street West, HSC-2C10B, Hamilton, Ontario, Canada

## Abstract

**Background:**

Economic evaluations in the medical literature compare competing diagnosis or treatment methods for their use of resources and their expected outcomes. The best evidence currently available from research regarding both cost and economic comparisons will continue to expand as this type of information becomes more important in today's clinical practice. Researchers and clinicians need quick, reliable ways to access this information. A key source of this type of information is large bibliographic databases such as EMBASE. The objective of this study was to develop search strategies that optimize the retrieval of health costs and economics studies from EMBASE.

**Methods:**

We conducted an analytic survey, comparing hand searches of journals with retrievals from EMBASE for candidate search terms and combinations. 6 research assistants read all issues of 55 journals indexed by EMBASE for the publishing year 2000. We rated all articles using purpose and quality indicators and categorized them into clinically relevant original studies, review articles, general papers, or case reports. The original and review articles were then categorized for purpose (i.e., cost and economics and other clinical topics) and depending on the purpose as 'pass' or 'fail' for methodologic rigor. Candidate search strategies were developed for economic and cost studies, then run in the 55 EMBASE journals, the retrievals being compared with the hand search data. The sensitivity, specificity, precision, and accuracy of the search strategies were calculated.

**Results:**

Combinations of search terms for detecting both cost and economic studies attained levels of 100% sensitivity with specificity levels of 92.9% and 92.3% respectively. When maximizing for both sensitivity and specificity, the combination of terms for detecting cost studies (sensitivity) increased 2.2% over the single term but at a slight decrease in specificity of 0.9%. The maximized combination of terms for economic studies saw no change in sensitivity from the single term and only a 0.1% increase in specificity.

**Conclusion:**

Selected terms have excellent performance in the retrieval of studies of health costs and economics from EMBASE.

## Background

With the rising costs of new technology for the diagnosis and management of disease, data concerning cost effectiveness of health care has become increasingly important, particularly for policy makers and managers of health services when making resource allocation decisions. It is also important that health professionals determine if the benefits will be worthwhile when considering the consumption of health care resources [1-3].

Economic evaluations in the medical literature compare competing diagnosis or treatment methods for their use of resources and their expected outcomes. Several databases provide access to this literature; some are specialty databases such as the U.K. National Health Service Economic Evaluation Database while others are large general purpose biomedical databases. End-users frequently access the medical literature online via the huge biomedical databases such as MEDLINE and EMBASE. Unfortunately, gaining access to this economic and cost literature through these databases can be daunting. The retrieval of relevant information is difficult due to the millions of articles and thousands of journals indexed, the minuscule concentration of articles with economic content, and the inconsistency of indexing within the databases [3,4].

Researchers have worked to develop search strategies to aid health professionals in the retrieval of relevant information, but this has been mostly for studies of treatment and diagnosis. Few reports exist of empirically validated search strategies for economic analyses and cost studies. Sassi et al [5] examined search strategies development within MEDLINE and our group recently reported high performance search strategies for MEDLINE which currently can be used when searching via PubMed from the National Library of Medicine website [6]. EMBASE indexes many journals that are not included in MEDLINE but, to the best of our knowledge, no studies have validated search strategies for economic analyses and cost studies in EMBASE.

In this paper, we report on search strategy development and retrieval performance for cost and economic studies indexed in EMBASE.

## Methods

The study compared the retrieval performance of methodologic search terms and phrases in EMBASE with a manual review of each article for each issue of 55 journal titles for the year 2000. Index terms and textwords related to research design features were run as search strategies. The search strategies were treated as "diagnostic tests" for sound studies and the manual review of the literature was treated as the "gold standard." The sensitivity, specificity, precision, and accuracy of EMBASE searches were determined. Sensitivity for a given topic is defined as the proportion of high quality articles for that topic that are retrieved; specificity is the proportion of low quality articles not retrieved; precision is the proportion of retrieved articles that are of high quality; and accuracy is the proportion of all articles that are correctly classified.

Six research assistants hand searched journals, and applied methodologic criteria to each item in each issue to determine if the article was methodologically sound for 7 purpose categories, including cost and economic evaluations, treatment, diagnosis, prognosis, clinical prediction, etiology, and reviews. Research staff was rigorously calibrated before reviewing the 2000 literature and inter-rater agreement for application of all criteria exceeded 80% beyond chance [7]. Cost studies and qualitative studies were also classified but had no methodologic criteria applied. All purpose category definitions and corresponding methodologic criteria were outlined in a previous paper [7]. Articles were categorized as cost studies based on the following criteria: Content pertains directly to the costs or financing or economics of a health care issue. Economics studies formed a subset of cost studies and were evaluated for methodologic rigor as follows: The study question is a comparison of alternatives; alternative services or activities are compared on outcomes produced (effectiveness) and resources consumed (costs); evidence of effectiveness must be from a study of real patients that meets the criteria for diagnosis, treatment, quality improvement, or a systematic review article; effectiveness and cost estimates are based on individual patient data (micro-economics); results are presented in terms of the incremental or additional costs and outcomes of one intervention over another; and sensitivity analysis is provided if there is uncertainty.

The 55 journals were chosen based on having the highest yield of methodologically sound articles across all purpose categories from a larger collection of 170 journal titles chosen based on recommendations of clinicians and librarians, Science Citation Index Impact Factors provided by the Institute for Scientific Information, and ongoing assessment of their yield of studies and reviews of scientific merit and clinical relevance for the disciplines of internal medicine, general medical practice, mental health, and general nursing practice (list of journals provided by the authors upon request). A total of 135 of the 170 journals were indexed in EMBASE, including the 55 top yielding journals used for this report. We had previously found that the developed search strategies were robust in smaller journal subsets [8] and that computation time was substantially decreased. We also found that when strategies were developed in 60% of the database and validated in the remaining 40% there were no statistical differences in performance. Thus, search strategies for EMBASE were developed using all data for 27,769 articles from 55 journals.

An initial list of index terms and textwords was compiled. Input was then sought from clinicians and librarians in the United States and Canada through interviews of known searchers, and requests at meetings and conferences. Individuals were asked to identify which terms or phrases they used when searching for studies of economics, costs, treatment, causation, diagnosis, prognosis, clinical prediction guides, reviews, and studies of a qualitative nature. We compiled a list of 5385 terms of which 4843 were unique and 3524 returned results (list of terms tested provided by the authors on request). Examples of the search terms relevant to costs and economics included 'cost effective', 'incremental costs', 'direct cost', and 'net benefit', all as textwords; 'cost effectiveness analysis', the index term, and the index term 'health economics', exploded.

The strategies for economic studies were tested for their ability to retrieve articles about high quality economics studies from all other articles, including both low quality economics studies and all non-economics studies. For costs studies, the strategies were tested for their discrimination between costs studies and all others. Individual terms with sensitivity > 25% and specificity > 75% for a given purpose category were incorporated into the development of search strategies that included a combination of 2 or more terms. All combinations of terms used the Boolean OR, for example, "effectiveness OR economics". The Boolean AND was not used because this strategy invariably compromised sensitivity. For the development of multiple-term search strategies to either optimize sensitivity or specificity, we tested all 2-term search strategies with sensitivity at least 75% and specificity at least 50%. For optimizing accuracy, 2-term search strategies with accuracy > 75% were considered for multiple-term development.

## Results

Indexing information was downloaded from EMBASE for 27,769 articles from the 55 hand-searched journals. Of these 183 were classified as about costs and 148 were classified as economics (a subset of costs studies). Of the economics studies, 31 (20.9%) were rated methodologically sound. A total of 40,116 search strategies were tested in the development of economics hedges and 16,728 for the development of the costs hedges.

Table 1 shows the best single term for high-sensitivity, high-specificity, and best balance of sensitivity and specificity. When maximizing sensitivity for detecting cost studies, the single term "exp economic aspect" produced the highest sensitivity at 98.9% with a specificity of 93.1%. For economics studies the single term "cost:.tw." produced the best sensitivity of 96.8% while achieving a specificity of 97.5%. When the specificity for cost studies was maximized, the single term "cost effective:.tw." produced a specificity of 99.4% but this was achieved at the expense of sensitivity, falling to 54.1%. Likewise, the maximization of specificity for economics studies, using the same single term of "cost effective:.tw.", resulted in a value of 99.2% but the sensitivity dropped to 64.5%. The optimal balance between specificity and sensitivity for a single term was achieved by "cost:.tw." for both cost studies and economics studies. For cost studies, this term produced a sensitivity of 96.2% and a specificity of 98.0% while for economics studies, it produced a sensitivity of 96.8% and a specificity of 97.5%.

Combination of terms with the best results for sensitivity, specificity and optimization of sensitivity and specificity are shown in Table 2. It was found that by combining terms, the sensitivity of searches for both cost studies and economics studies could achieve 100% sensitivity. For cost studies, the 2-term search strategy of "exp economic aspect OR costs.tw." resulted in a sensitivity of 100.0% and a specificity of 93.0%. For economics studies, the 3-term search strategy of "cost effectiveness analysis.sh. OR randomized.tw. OR economic.tw." also produced a sensitivity of 100.0% but with a slightly lower specificity of 92.3%. However, in comparison to the single term results, the combination of terms did not prove more successful when maximizing for specificity. For cost studies, the 2-term search strategy of "cost effectiveness.tw. OR cost effective.tw." produced a specificity of 99.5% and a sensitivity of 54.1%, a result which improved on the single term strategy by only 0.1% for specificity. For economics studies, the 2-term search strategy of "cost effectiveness.tw. OR sensitivity analys:.tw." resulted in a specificity of 99.4% and a sensitivity of 51.6%, a 0.2% specificity increase over the single term strategy but a 12.9% drop in sensitivity. When optimizing for sensitivity and specificity, the combined strategies for the cost studies produced slightly more precise results than the single term strategies. The optimized combination search strategy of "cost.mp. OR costs.tw. OR health care cost.sh." resulted in a sensitivity of 98.4% and a specificity of 97.1%. For economics studies, the combination strategy of "cost.tw. OR costs.tw." produced an optimized result of 96.8% sensitivity and 97.6% specificity which is only a 0.1% improvement for specificity over the single term strategies.

## Discussion

This investigation shows that selected single terms and combinations of search terms can reach high levels of performance in the retrieval of high quality literature in the area of economic analysis and in the retrieval of literature focusing on the cost of health care services. By assisting in the retrieval of relevant cost literature, clinicians and researchers will be able to find the information they need more dependably and quicker, perhaps improving evidence-based decisions. Single term and combination search strategies have been shown to be highly sensitive and specific in the areas of cost and economics. Although there was little difference when comparing single and combination strategies for cost articles, the economics articles saw much better performance in terms of sensitivity for the single term strategy than the combination strategy when optimizing for specificity. Finally, when optimizing for both specificity and sensitivity, the combination strategies for the cost searches saw a slightly more sensitive return than the single strategies, while there was no real difference when comparing the type of search strategies when applied to the economics searches.

It is important to note that several top performing terms are exploded index terms and many are text words. In the event that new index terms relevant to cost and/or economic studies are added to Emtree, it is likely that our reported search strategies will perform similarly in terms of sensitivity and specificity. Text word searching involves only the title and abstract of the article so additions to Emtree will have no effect on the performance of these terms. Additionally, if new index terms are added and if they are closely related to the exploded index term included in the search strategy, the articles indexed with the new term will be retrieved.

In all points of comparison, the investigated search strategies performed well in terms of the accuracy of their returns. In fact, all accuracy values were over 92%. Even so, the precision of searches, that is, the proportion of retrieved articles that are on target, is suboptimal. This is simply a reflection of the very low concentration of cost and economics in the huge EMBASE database; for sound economics studies, the concentration was less than 0.1%. Precision is dependent on the concentration of target articles (in this case, cost or economic studies) in the entire database. We tested our search strategies in a subset of EMBASE records. Therefore, the precision figures reported are included only as an illustration of search strategy performance. When searching in the entire EMBASE database, precision will be lower.

While two of the single term cost strategies achieved precision levels of 39.4% and 24.0%, none of the economics single term strategies achieved better than 8% precision, meaning that only 8% of the retrieved articles were on target. The overall precision decreased further for the combination search strategies when compared to the single term strategies. Thus the somewhat higher sensitivity of the combination strategies is at the expense of decreased precision and accuracy. In addition to precision being dependent on the concentration of target articles in the entire database, low precision returns could also point to a potential problem of over indexing, that is, index terms that appear to be specific to good quality economic studies are not used solely for those types of articles resulting in the retrieval of many false positive articles (i.e., studies that are not evaluating the cost or economics of a health care situation).

Finally, the methodologic criteria for economic studies are fairly rigorous as noted by the low number of pass economic studies (n = 31) in the database. Since pass and fail economic studies are a subset of cost studies, searchers could use the cost strategies if they fail to find relevant articles when searching using the economic strategies. This is also true for economic studies based on models. Our definition of a pass economic study required that the study be based on data from real patients. Therefore, those that were based on models   would only be retrieved as "false positives" when using our economic search strategies but have high likelihood of being retrieved when using the cost strategies.

We recently published economics and cost search strategies to use when searching in MEDLINE in the context of retrieving literature relevant to health services research (HSR) [6]. The HSR strategies were developed in a subset of journals (n = 68) that are indexed in MEDLINE and that publish HSR literature (there is some overlap with the EMBASE journal list). When comparing the EMBASE search strategies with those reported for use in MEDLINE we find some similarities. Optimal search strategies for use in EMBASE and MEDLINE are made up of both index terms and text words. Cost.tw. and/or a variation thereof (e.g., costs.tw., cost:.mp) is a top performer in both databases as is cost effectiveness.tw. and/or a variation thereof (e.g., cost effective:.tw.). Sensitivity analys:.tw. is a top performer in both EMBASE and MEDLINE when performing a highly specific search for economic articles. The index terms that were top performers in EMBASE and MEDLINE are quite different. This difference is partially due to the fact that some of the top performing index terms are not supported in the other database. For example, the index term "cost effectiveness analysis" is a top performer in EMBASE but this is not an index term in MEDLINE. Overall, although the search strategies developed for EMBASE and MEDLINE are different there are many similarities when comparing the text words that are top performers.

Multivariate statistical techniques may yield better results than we observed. However, when we tested a logistic regression approach to developing search strategies for MEDLINE, we found no improvement on the same Boolean approach used in the EMBASE study [9]. Even if such techniques did improve yield, the increase would be marginal at best, given how well the strategies shown here work, except perhaps for increasing sensitivity for strategies optimizing specificity. Such strategies would also likely have the disadvantage of being more complex, and thus harder to implement.

Machine learning methodologies may yield better results than we observed. We are currently exploring this possibility through collaborative ventures with two research groups in the United States.

## Conclusion

By combining specific textwords and terms with  multiple postings (.mp.), one can greatly improve the retrieval of costs and economics literature from EMBASE.

## Competing interests

The author(s) declare that they have no competing interests.

## Pre-publication history

The pre-publication history for this paper can be accessed here:



## Figures and Tables

**Table 1:** Single term with the best sensitivity (keeping specificity ≥50%), best specificity (keeping sensitivity ≥50%), and best optimization of sensitivity and specificity (based on the lowest possible absolute difference between sensitivity and specificity) for detecting costs and economics studies in EMBASE in 2000. Values are percentages (95% CIs).

Search term OVID search*	**Sensitivity (%) (Cost: n = 183; Economics: n = 31)**	**Specificity (%) (Cost: n = 27586; Economics: n = 27738)**	**Precision (%)†**	**Accuracy (%) (n = 27769)**
**Best sensitivity – Costs **exp economic aspect	98.9 (97.40 to 100.41)	93.1 (92.84 to 93.44)	8.7 (7.51 to 9.94)	93.2 (92.88 to 93.47)
**Best sensitivity – Economics **cost:.tw.	96.8 (90.55 to 102.99)	97.5 (97.31 to 97.67)	4.1 (2.68 to 5.58)	97.5 (97.31 to 97.67)
**Best specificity – Costs **cost effective:.tw.	54.1 (46.88 to 61.32)	99.4 (99.36 to 99.54)	39.4 (33.40 to 45.49)	99.2 (99.04 to 99.26)
**Best specificity – Economics **cost effective:.tw.	64.5 (47.67 to 81.36)	99.2 (99.06 to 99.28)	8.0 (4.64 to 11.36)	99.1 (99.02 to 99.24)
**Best Optimization of Sensitivity & Specificity – Costs **cost:.tw.	96.2 (93.40 to 98.95)	98.0 (97.82 to 98.15)	24.0 (20.95 to 27.14)	98.0 (97.81 to 98.14)
**Best Optimization of Sensitivity & Specificity – Economics **cost:.tw.	96.8 (90.55 to 102.99)	97.5 (97.31 to 97.67)	4.1 (2.68 to 5.58)	97.5 (97.31 to 97.67)

*The search strategy is reported using Ovid's search engine syntax for EMBASE. †Denominator varies by row. exp = exploded subject heading; : = truncation; tw = textword (word or phrase appears in title or abstract).

**Table 2:** Combination of terms with the best sensitivity (keeping specificity ≥50%), best specificity (keeping sensitivity ≥50%), and best optimization of sensitivity and specificity (based on abs[sensitivity-specificity]<1%) for detecting costs and economics studies in EMBASE in 2000. Values are percentages (95% CIs).

Search term OVID search*	**Sensitivity (%) (Cost: n = 183; Economics: n = 31)**	**Specificity (%) (Cost: n = 27586; Economics: n = 27738)**	**Precision (%)†**	**Accuracy (%) (n = 27769)**
**Best Sensitivity – Costs **exp economic aspect OR costs.tw.	100.0	93.0 (92.68 to 93.29)	8.6 (7.44 to 9.83)	93.0 (92.73 to 93.33)
**Best Sensitivity – Economics **cost effectiveness analysis.sh. OR randomized.tw. OR economic.tw.	100.0	92.3 (91.98 to 92.61)	1.4 (0.93 to 1.93)	92.3 (91.99 to 92.62)
**Best Specificity – Costs **cost effectiveness.tw. OR cost effective.tw.	54.1 (46.88 to 61.32)	99.5 (99.37 to 99.54)	39.8 (33.68 to 45.84)	99.2 (99.05 to 99.26)
**Best Specificity – Economics **cost effectiveness.tw. OR sensitivity analys:.tw.	51.6 (34.02 to 69.21)	99.4 (99.31 to 99.49)	8.8 (4.68 to 12.91)	99.4 (99.25 to 99.44)
**Best Optimization of Sensitivity & Specificity – Costs **cost.mp. OR costs.tw. OR health care cost.sh.	98.4 (96.52 to 100.20)	97.1 (96.87 to 97.27)	18.2 (15.81 to 20.63)	97.1 (96.88 to 97.28)
**Best Optimization of Sensitivity & Specificity – Economics **cost.tw. OR costs.tw.	96.8 (90.55 to 102.99)	97.6 (97.43 to 97.79)	4.3 (2.82 to 5.85)	97.6 (97.43 to 97.79)

*Search strategies are reported using Ovid's search engine syntax for EMBASE. †Denominator varies by row. exp = exploded subject heading; tw = textword (word or phrase appears in title or abstract); : = truncation; mp = multiple posting – term appears in title, abstract, or subject heading; sh = subject heading.
